# Real-Time Observations of Food and Fluid Timing During a 120 km Ultramarathon

**DOI:** 10.3389/fnut.2018.00032

**Published:** 2018-05-04

**Authors:** Floris C. Wardenaar, Daan Hoogervorst, Joline J. Versteegen, Nancy van der Burg, Karin J. Lambrechtse, Coen C. W. G. Bongers

**Affiliations:** ^1^School of Nutrition and Health Promotion, College of Health, Arizona State University, Phoenix, AZ, United States; ^2^Sports and Exercise Nutrition, Institute for Sports and Exercise, HAN University of Applied Sciences, Nijmegen, Netherlands; ^3^Global Nutrition Development, FrieslandCampina, Amersfoort, Netherlands; ^4^Radboud Institute for Health Sciences, Radboud University Medical Center, Nijmegen, Netherlands

**Keywords:** running, sports nutrition, recommendations, supplements, sweat rate, fluid balance

## Abstract

The aim of the present case study was to use real-time observations to investigate ultramarathon runners' timing of food and fluid intake per 15 km and per hour, and total bodyweight loss due to dehydration. The study included 5 male ultramarathon runners observed during a 120 km race. The research team members followed on a bicycle and continuously observed their dietary intake using action cameras. Hourly carbohydrate intake ranged between 22.1 and 62.6 g/h, and fluid intake varied between 260 and 603 mL/h. These numbers remained relatively stable over the course of the ultra-endurance marathon. Runners consumed food and fluid on average 3–6 times per 15 km. Runners achieved a higher total carbohydrate consumption in the second half of the race (*p* = 0.043), but no higher fluid intake (*p* = 0.08). Energy gels contributed the most to the total average carbohydrate intake (40.2 ± 25.7%). Post-race weight was 3.6 ± 2.3% (range 0.3–5.7%) lower than pre-race weight, revealing a non-significant (*p* = 0.08) but practical relevant difference. In conclusion, runners were able to maintain a constant timing of food and fluid intake during competition but adjusted their food choices in the second half of the race. The large variation in fluid and carbohydrate intake indicate that recommendations need to be individualized to further optimize personal intakes.

## Introduction

An ultramarathon is any footrace longer than the traditional marathon length of 42.195 km. Ultra-endurance performance is highly dependent on the intake of carbohydrates during the race. In addition to muscle glycogen stores, up to 60–90 g of exogenous carbohydrates per hour are advised to maintain relatively high exercise intensity levels during ultra-endurance performance (1–5). Carbohydrate oxidation in recreational well-trained, non-fat adapted runners has shown to be the dominant energy source (≥70% of the total energy expenditure) (6, 7). Previous studies investigating nutrient intake in ultra-endurance events reported a wide range of carbohydrate intake (23–71 g/h), which was often lower than the recommended intake of 60–90 g carbohydrates per hour (8–19).

Besides the need to consume sufficient carbohydrates, ultramarathon runners need to prevent excessive dehydration (>2% body weight loss from water deficit) particularly in prolonged exercise lasting greater than 3 h (20). Previously reported fluid intake in ultra-runners was 354–765 mL/h (8, 10, 11, 13–19, 21). In some of these studies, fluid balance was measured by indication of pre and post-race body weight, but actual sweat rate estimation was not reported. In other sports, with a shorter duration (i.e., waterpolo, netball, swimming, rowing, basketball, soccer, American football, tennis, and running), sweat rate estimates range from ~0.3 to ~1.8 L/h (20). Overall, it can be debated if fluid intake during previous ultramarathons was optimal or could be improved.

Several factors might limit the carbohydrate and fluid intake over the course of the ultra-endurance running event. These factors may include, but are not limited to, environmental conditions (i.e., temperature, humidity) (14), exertion as a result of competition, pacing strategy, restricted feeding options during competition (22), the aid stations design (i.e., placing, frequency, and product range), and gastro-intestinal (GI) distress (14). Many ultra-endurance runners experience gastro intestinal complaints during an ultra-endurance competition, which may complicate the regular ingestion of nutrients and fluid during the race (17, 23). GI distress can be divided by upper and lower GI complaints. Both the upper and lower GI complaints likely have different mechanisms, but they all limit feeding as a result of discomfort (24). Interestingly, we previously demonstrated that a large set of GI complaints (i.e., heartburn, belching, vomiting, intestinal cramp, urge to defecate, side ache, loss of stool, and diarrhea) are negatively correlated with the intake of energy, carbohydrate, and fluid (17).

Food and nutrient intake during ultra-marathons has often been self-reported using a questionnaire or recalls (15, 18, 19, 25). However, recalling all foods and drinks ingested during a daylong ultra-endurance event is difficult, thereby hampering the validity of the results. Moreover, most studies only reported total food and nutrient intake, rather than the timing of food, and nutrient intake over the course of the ultra-endurance event.

Although sufficient intakes of carbohydrates and fluid are essential for ultra-endurance performance, actual intakes are the result of the timing and amounts of food and fluid consumed during competition. So far, only one *n* = 1 female case study by Moran et al. (13) reported timing of food and fluid during an ultramarathon. Because feeding strategies differ between athletes, more observations of timing of food and fluid intake are needed. Therefore, the aim of the present study was to use real-time observations to investigate whether ultramarathon runners were able to maintain constant intakes of carbohydrates and fluid to meet recommendations over the course of a 120 km ultramarathon.

## Materials and methods

### Study design

This cross-sectional study was designed to provide descriptive information using real-time observation on food and fluid intake, hydration status, and GI complaints in ultra-endurance athletes in April, 2017, in the Netherlands. The study was performed in accordance with the Declaration of Helsinki and was approved by the Ethical Advisory Board of the HAN University of Applied Sciences (EACO 63.03/17). Written informed consent was obtained from all runners.

### Study population

Runners registered for the 120 km distance of the Zestig van Texel (“Sixty of Texel”) event in the Netherlands. All runners included in this study completed a short web-based questionnaire about their personal characteristics, general health and medical condition, and running history. Nine healthy male runners without injuries, from 18 to 55 years old (47 ± 6 years), participated. Each of the runners had previously completed at least 10 (ultra)marathons during their running career. Two of the runners said to use a carbohydrate rich diet in the days before the race, one of the runners said to focus mainly on extra fluid intake and five runners did not change their dietary intake in preparation for the race day. One runner said to follow a high fluid and carbohydrate rich diet in the days before the race.

### Procedures

All selected runners received extensive information at least 5 days before the race. An interview was scheduled for the day before the race with all included runners. During the interview, the food and beverage labeling and product registration was explained. The organization of this ultramarathon allowed runners to be cared for by a personal cyclist that could hand out beverage and food products throughout the whole race. The most important reason for this is runners of the 120 km cover two sections of 60 km, but during the first 60 km no aids stations were available. Runners brought their own products and were allowed to have their own nutrition strategy. They labeled their own products with a unique code that was provided to them, and a research team member double-checked whether this was done correctly. Next, all products were weighed, including the original wrapping when relevant. Pictures were taken of all foods and beverages, including labels and ingredient declarations.

On the day of the race, body weight and height (Cescorf stadiometer, Porto Alegre, Brazil) were measured (without shoes in light racing clothes) 1 h before the start (5.35 a.m.). Runners were asked to empty their bladder before measuring body weight. Thereafter, all food and fluid intake was observed and urine excretion collected until completing the ultramarathon. During the race, the runners were accompanied by a research team member on a bicycle for continuous observation of nutritional intake and urine excretion. The member recorded the time for each 15 km milestone. Runners reported GI complaints, as well as their rating of perceived exertion (RPE) using a 6–20 categorical Borg scale. Directly after completing the race, body weight, total urine excretion, and the weight difference of all consumed products were measured.

### Dietary intake

#### Observation of intake

Action cameras were attached to the bicycles (SJCAM, SJ4000, Shenzhen, China) to record all food and fluid consumptions. During the registration, the observant reaffirmed with the runner, on camera, all consumed products and beverages, each 15 km milestone time (hh:mm:ss), RPE and GI complaints.

#### Products

Food products and fluids, including wrapping or wrapping only, were measured on a digital scale (Cresta, CKS750, Amsterdam, The Netherlands) before and after the race with 0.1-g accuracy. The difference between measurements was calculated to obtain the actual consumed amount of each product (g). In addition, all foods were categorized as: sports drink (isotonic and hypertonic formulas), gels, cola, chocolate milk, water, other fluid (all other drinks consumed), other solid (all other products consumed), and bars.

#### Nutrients

Based on the total product weight and ingredient declarations, the total consumed amount of energy (kcal); grams (g) of carbohydrate, protein, fiber and fat; and milliliters of fluid (mL) were calculated.

### Fluid balance

#### Body weight difference

Body weight (Seca scale S760 mechanical, Hamburg, Germany) was measured pre- and post-race. Furthermore, the absolute and relative body weight loss (difference between pre- and post-race) was calculated, in which the relative body mass is presented as dehydration level (%).

#### Urine collection

The runners were instructed to urinate into a specialized collecting bag (Roadbag, KETs GmbH, Köln, Germany) from 60 min pre-race until directly after the race to determine the total urine excretion. The urine bags were provided and collected as needed at all points during the race. However, due to the inability to cycle at the beach sections of the race, it was not possible to collect urine (24.5 km total). During the race, only one urine excretion that took place on the beach section was not collected. For this runner, an estimated amount of 125 ml was added to his total collected urine excretions to calculate total urine loss. The urine bags were weighted after the race with a 0.1 g accuracy (PT 1400, Sartorius AG, Göttingen, Germany).

#### Sweat rate

The estimated sweat rate was calculated as total weight lost, corrected for all consumed foods and beverages and collected urine during the race (as: weight loss (kg) + Fluid and beverages consumed in kg–total urine loss in kg). As respiratory water losses and the production of metabolic water as a result of cellular metabolism are approximately equal, these were not included in this equation (20).

### Measurements per 15 Km

#### RPE and split time

Within 200 m before each 15 km milestone, runners were asked to appoint their rating perceived exertion (RPE). Before the race, they were familiarized with a 6–20 categorical Borg scale, in which 6 was very light and 20 very hard. Furthermore, at every 15 km milestone, the split time was noted, which was used to examine the pacing strategy of the runners.

#### GI distress

The runners were instructed to report GI complaints using a questionnaire at every 15 km milestone. The GI complaints were assessed using a previously described pre-specified list of complaints, which was also used prior to and directly after finishing the race. After the race, runners were asked to fill out, within 24 h, a previously described questionnaire (17) in which runners identified again all GI complaints they experienced over the race. In both cases, the runners were instructed to rank the specific identified GI complaints on a nine-point scale (“no problem at all” to “the worst it has ever been”). If a complaint had a score >4, it was considered serious as described previously by Pfeiffer et al. (14).

### Data analysis

All calculations were performed in Excel (2016) and SPSS (IBM SPSS Statistics, version 23). Characteristics of runners—i.e., finish time (hh:mm), speed (km/h), height (cm), weight (kg), consumption moments, combined food and fluid intake (kg), urine excretion (L), sweat rate (L), and absolute and relative body weight loss (kg and %)—were reported as individual and group data (mean±sd). Total consumed food and fluid per runner and for the group (mean±sd) were expressed as total energy (kcal), carbohydrates, protein, fiber (in g/h), and fluid (mL/h). The average CHO intake per hour was expressed per food group for each individual. The total amount of consumed foods per category (g) were expressed per 15 km milestone, as well as nutrient intake (CHO, PRO, fluid, fiber, fat), pacing (min/km), RPE, consumption moments, and urine excretion (g). GI complaints during the race and GI complaints based on the questionnaire were reported as descriptive (incidence and score).

Difference between pre-race and post-race weight and difference between running speed and average nutrient intake per 15 km or per hour during the first and the second part of the race were tested based on the Wilcoxon Signed Rank test, with the level of significance set at *p* ≤ 0.05.

## Results

### Exercise and environmental conditions

Complete datasets were collected for only 5 of the subjects, as 3 runners withdrew from the race at the 60 km turning point and 1 runner dropped out earlier because of an injury. Runners completed the 120 km distance with an average speed of 9.9 ± 1.5 km/hour, as shown in Table 1. Running speed during the first part of the race was higher than in the second part, which resulted in a faster first 60 km compared to the second 60 km (05:32 ± 00:30 h vs. 06:47 ± 01:01 h, respectively, *p* = 0.043). The RPE at start was 6.2 ± 0.4 vs. a RPE at finish of 16.2 ± 1.9). The average temperature on the day of the race was 7.0°C (min: 4.3°C and max: 9.6°C). Humidity was 67% with 1.6 h of rain and 11.1 h of sunshine between sunrise and sunset. The average wind speed was 5.0 m/s (measured at weather station De Kooy, Den Helder).

**Table 1:** Characteristics of ultramarathon runners during 120 km race (*n* = 5).

**Runner**	**Finish time (hh:mm)**	**Average speed (km/h)**	**Height (cm)**	**Pre-race weight (Kg)**	**Post-race weight (Kg)**	**Consumption moments**	**Food/fluid intake (Kg)**	**Urine excretion (L)**	**Fluid loss, sweat rate (L)**	**Absolute body weight loss (Kg)**	**Level of dehydration (%)**
1	09:50	12.2	184	71.5	69.0	48	4.7	1.1	6.0	2.5	3.5
2	11:26	10.5	178	65.5	61.8	36	4.2	0.5	7.5	3.8	5.7
3	13:08	9.1	185	78.0	74.5	48	7.9	0.3	10.1	3.5	4.5
4	13:20	9.0	187	75.0	75.2	25	5.3	0.6	4.6	−0.2	0.3
5	13:49	8.7	178	65.8	62.7	31	3.8	1.4	4.9	3.1	4.6
Mean ± *SD*	12:19 ± 01:29	9.9 ± 1.5	182 ± 4	71.2 ± 5.5	68.6 ± 6.3	37.6 ± 10.3	5.4 ± 1.7	0.8 ± 0.5	6.6 ± 2.3	2.5 ± 1.6^*^	3.6 ± 2.3

* *No significant difference between pre-race and post-race weight based on Wilcoxon Signed Rank Test, p = 0.08*.

### Food and carbohydrate intake

**Figure 1:**
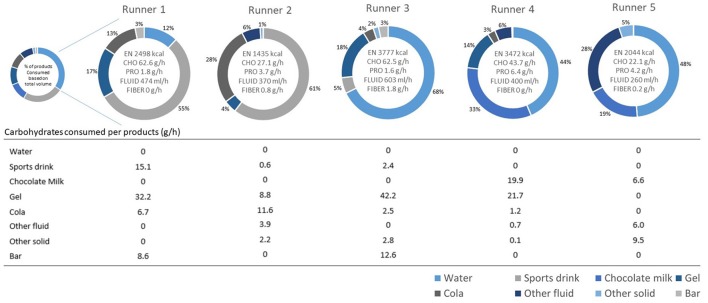
Total energy and nutrient intake per hour, percentage (%) volume of total consumed food groups, and carbohydrate intake (CHO) per food group in g/h per runner.

Total energy and nutrient intake varied substantially between the five runners. Each runner consumed products from 3 to 5 of the previously defined food categories (Figure 1). Total energy intake during the race varied between 1,435 and 3,777 kcal. The combined total food and fluid intake was 5.4 ± 1.7 kg with a total of 0.2 ± 0.2 kg of solid foods. Carbohydrates were mainly delivered as part of fluids. Total mean fluid-based carbohydrate intake was 440 ± 193 vs. 91 ± 83 g of carbohydrates delivered by solid foods. Energy gels contributed the most to the total carbohydrate intake (43.6 ± 19.1 g/h). Individual carbohydrate intake ranged between 22.1–62.6 g/h, and fluid intake (421±127 mL/h) varied between 260–603 mL/h. As shown in Table 2, product choices in the first 60 km of the race were very consistent (i.e., consumption of energy gel, water, and sport drink). During the second part of the race other types of foods were introduced to the pattern. Although water and sports drinks remained the most important energy and fluid sources during the second part, chocolate milk and cola also contributed. Overall a less structured pattern was seen in the second part of the race in comparison to the first part of the race.

**Table 2:** Consumed products per 15 km (volume in g) and most used products on group level (person count).

	**0–15 km**	**15–30 km**	**30–45 km**	**45–60 km**	**60–75 km**	**75–90 km**	**90–105 km**	**105-−120 km**
Runner 1	Sport drink (518 g)	Sport drink (259 g)	Sport drink (389 g)	Sport drink (259 g)	Sport drink (289 g)	Sport drink (259 g)	Cola (450 g)	Cola (150 g)
	Sport bar (43 g)	Gel (141 g)	Gel (72 g)	Gel (63 g)	Gel (77 g)	Gel (148 g)	Sport drink (389 g)	Gel (131 g)
	Gel (77 g)	Sport bar (22 g)	Sport bar (43 g)	Sport bar (22 g)	Sport bar (22 g)	Water (138 g)	Water (330 g)	Sport drink (130 g)
							Gel (122 g)	Water (100 g)
Runner 2	Sport drink (539 g)	Sport drink (606 g)	Sport drink (439 g)	Sport drink (414 g)	Sport drink (138 g)	Sport drink (276 g)	Cola (300 g)	Cola (750 g)
	Gel (78 g)	Gel (109 g)	Gel (132 g)	Gel (138 g)		Cola (150 g)	Sport drink (230 g)	
						Other solid (47 g)		
						Sweets (11 g)		
Runner 3	Gel (355 g)	Water (517 g)	Water (430 g)	Water (602 g)	Water (1,016 g)	Water (844 g)	Water (974 g)	Water (946 g)
	Water (230 g)	Gel (174 g)	Gel (267 g)	Other solid (54 g)	Gel (163 g)	Gel (347 g)	Gel (153 g)	Sport drink (300 g)
		Other solid (67 g)			Other solid (109 g)	Sport drink (150 g)	Other solid (62 g)	Cola (300 g)
						Fruit (83 g)	Fruit (17 g)	Fruit (33 g)
Runner 4	Water (446 g)	Water (408 g)	Water (676 g)	Gel (108 g)	Choc. milk (418 g)	Choc. milk (361 g)	Choc. milk (779 g)	Choc. milk (256 g)
	Gel (160 g)	Gel (160 g)	Gel (331 g)	Water (78 g)		Water (349 g)	Other fluid (330 g)	Water (229 g)
						Cola (150 g)	Water (182 g)	
						Fruit (20 g)		
Runner 5	Water (120 g)	Water (360 g)	Water (250 g)	Other fluid (256 g)	Choc. milk (245 g)	Water (983 g)	Other fluid (337 g)	Choc. milk (261 g)
		Other solid (28 g)	Other solid (41 g)	Water (250 g)		Other fluid (256 g)		Other fluid (254 g)
				Other solid (70 g)		Choc. milk (248 g)		
						Other solid (70 g)		
#1 used products	Gel (4)	Gel (4)	Gel (4)	Water (3)	Choc. milk (2) Sport drink (2)	Water (4)	Water (3)	Cola (3) Water (3)
#2 used products	Water (3)	Water (3)	Water (3)	Gel (3)	Gel (2)	Sport drink (3)	Cola (2)	Sport drink (2)
							Sport drink (2)	Choc. milk (2)
							Gel (2)	
							Other fluid (2)	
#3 used products	Sport drink (2)	Sport drink (2)	Sport drink (2)	Sport drink (2)		Choc milk (2)		
		Other solid (2)		Other solid (2)		Cola (2) Gel (2) Fruit (2) Other solid (2)		

### Fluid intake and estimated sweat rate

Post-race body weight was lower in 4 out of 5 runners (Table 1). Total fluid intake, based on the consumption of liquids, was 5.1 ± 1.6 L, which was lower than a total estimated sweat rate of 6.6 ± 2.3 L. The average urine excretion was 0.8 ± 0.5 L. Furthermore, the level of dehydration of the runners after completing the race was 3.6 ± 2.3% (range 0.3–5.7%), which was not significant (*p* = 0.08).

### Timing of food and fluid intake

Table 3 shows that runners had 3–6 consumption moments per 15 km during the race. The highest observed number of consumption moments during the race was observed in the second part of the race (after completing 75 km). In this 30 km period, between 75–90 and 90–105 km, the total number of consumption moments was 6.2 ± 1.1 and 5.6 ± 3.5 consumption, respectively. The larger total intake resulted also in the highest intakes of carbohydrates (90.6 ± 38.2 and 79.0 ± 43.9 g/h) and fluid (950 ± 429 and 907 ± 394 mL/h) during the race.

**Table 3:** Surface, Pacing, rate of perceived exertion, eating moments, fluid excretion, and nutrient intake per 15 km (*n* = 5).

	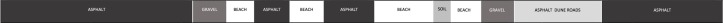							
	**0–15 km**	**15–30 km**	**30–45 km**	**45–60 km**	**60–75 km**	**75–90 km**	**90–105 km**	**105–120 km**
Pacing (min/km)	5:41 ± 0:32	5:21 ± 0:17	5:34 ± 0:32	5:38 ± 0:44	6:45 ± 1:24	6:57 ± 0:47	6:12 ± 0:46	7:08 ± 1:47
RPE	8.4 ± 2.1	9.6 ± 1.7	11 ± 2	12.4 ± 3.1	14.6 ± 2.7	15.2 ± 2.2	15 ± 3.8	16.2 ± 1.9
Eating moments	4.0 ± 2.1	4.6 ± 0.9	5.6 ± 1.8	3.8 ± 0.8	3.0 ± 2.8	6.2 ± 1.1	5.6 ± 3.5	4.8 ± 2.0
Urine excretion (mL)	144 ± 127	276 ± 218	59.0 ± 76.2	18.4 ± 36.8	25.0 ± 50.0	87.2 ± 149	69.9 ± 99.1	112 ± 92.3
CHO (g)	58.5 ± 48.8	60.5 ± 32.9	73.0 ± 38.1	48.2 ± 15.3	58.1 ± 42.7	90.6 ± 38.2	79.0 ± 43.9	61.2 ± 16.9
PRO (g)	2.1 ± 3.0	3.1 ± 2.9	2.5 ± 2.5	4.7 ± 3.8	8.3 ± 7.5	9.2 ± 7.3	10.5 ± 14.3	5.3 ± 6.0
Fluid (mL)	505 ± 193	551 ± 141	598 ± 247	430 ± 151	489 ± 374	950 ± 429	907 ± 394	766 ± 414
Fiber (g)	0.2 ± 0.4	1.9 ± 2.2	0.8 ± 1.0	0.6 ± 0.9	3.7 ± 3.8	4.6 ± 0.7	1.1 ± 2.1	0.1 ± 0.3
FAT (g)	0.1 ± 0.2	0.5 ± 0.5	0.4 ± 0.7	4.6 ± 5.5	6.5 ± 6.7	7.1 ± 8.2	7.8 ± 12.3	4.4 ± 5.4

*Surface is shown from the start (left side of the figure) until the finish after 120 km (right side of the figure), each column within the table represents a 15 km distance. All data represents mean values ± SD per 15 km*.

The pattern differed between the first and the second 60 km of the race. The absolute carbohydrate intake per 15 km was lower for the first 60 km of the race than the second part (60.1 ± 33.8 vs. 72.2 ± 35.4 g/15 km, *p* = 0.04). No difference for carbohydrate intake was seen when intake was divided in two equal time periods based on finish time (*p* = 0.69), as average carbohydrate intake was 46.9 ± 17.0 vs. 46.5 ± 14.1 g/h. No differences were detected for fluid intake between the first and second 60 km of the race (521 ± 183 vs. 778 ± 403 mL/15 km, *p* = 0.08). Likewise, no difference was seen for fluid intake between the first and second half of the race based on finish time (379 ± 89.0 vs. 461 ± 161 mL/h, *p* = 0.35).

### Gastro intestinal complaints

Runners reported 1–2 GI complaints during the race, but the type and frequency of complaints as well as the severity (score 3–8) of these complaints varied between runners (Table 4). Interestingly, during the race, runners reported a substantially lower amount of experienced complaints in comparison to the self-reported complaints in the post-race questionnaire (1–2 reported during competition vs. 3–8 self-reported after competition per person). Based on this questionnaire, all runners reported both upper and lower GI complaints. In the post-exercise questionnaire, runners reported reflux (*n* = 3), nausea (*n* = 2), belching (*n* = 2), and bloating (*n* = 3), which all might result in perceived discomfort. Finally, three out of five runners also reported an urge to urinate during the race, based on the questionnaire.

**Table 4:** Gastro intestinal complaints assessed during the race and based on a post-race questionnaire (*n* = 5).

	**During the race (assessed each 15 km)**			**Post-race (questionnaire)**		
	**Upper GI**	**Lower GI**	**Other**	**Upper GI**	**Lower GI**	**Other**
Runner 1			Dizziness (5;5) Urge to urinate (3)	Belching (2) Bloating (2) Nausea (2)	Flatulence (2)	Dizziness (5) Headache (2) Urge to urinate (4)
Runner 2		Flatulence (4;4;8)		Reflux (6) Bloating (6)	Flatulence (4)	
Runner 3	Nausea (3)			Nausea (3)	Urge to defecate (5)	Urge to urinate (4)
Runner 4		Side ache (6)		Reflux (9) Bloating (2)	Intestinal cramp (7) Flatulence (7) Urge to defecate (3) Side ache (8) Abdominal pain (2)	Urge to urinate (5)
Runner 5		Urge to defecate (5;5;4;3;3)		Reflux (4) Heartburn (4) Belching (5)	Intestinal cramp (4) Urge to defecate (5) Diarrhea (5)	

*Upper GI, Upper respiratory and stomach complaints like: reflux, heartburn, belching, bloating, stomach cramps, vomiting, and nausea. Lower GI, Lower bowel complaints like: intestinal cramp, flatulence, urge to defecate, side ache, abdominal pain, loose of stool, diarrhea, and intestinal bleeding. Other, Dizziness, headache, muscle cramps, urge to urinate. During the race runners could score each 15 km potential complaints, in case athletes reported a complaint multiple times this is indicated by multiple scores between brackets (X;X). Each complaint that was mentioned received a score >0. If a complaint had a score >4, it was considered as serious*.

## Discussion

The aim of the present study was to use real-time observations to investigate whether ultramarathon runners were able to maintain constant intakes of carbohydrates and fluid to meet recommendations over the course of a 120 km ultramarathon. Despite the fact that all runners experienced GI complaints during the event, the intake of carbohydrate and fluid per hour remained relatively stable during the race. The unique setting of this race, allowing the runners to have continuous feedings opportunities delivered by their accompanying cyclists, showed only small variations in CHO intake between runners for each 15 km covered. On the contrary runners were not able to constantly consume a high amount of carbohydrates and fluid intake was on average much lower than the estimated sweat rate.

Overall carbohydrate and fluid intake in the present study was lower than 2013 reports from other Dutch ultramarathon runners, where the observed intake was >30 g of carbohydrate and >350 mL of fluid per hour (17). On the other hand the reported average CHO and fluid intake of the present study fits within the range of earlier reports, reporting a carbohydrate intake 37.0–67.2 g/h and a fluid intake of 354–765 mL/h (10–13, 16, 17). These reports used observation or recall during the race instead of post-race questionnaires during different ultramarathons.

To our knowledge this is the first study in 20 years capturing urine excretion during competition (26). During the present data collection, urine excretion was, on average, 11% of the total weight lost vs. 4.6% urine loss reported in a multi-event study by Rogers et al. (26). The focus of the multi-event study was to assess fluid balance, including urine excretion, at a higher temperature than the current study (7 vs. 28°C). This temperature difference might explain a part of the disparity in the percentage of urine loss between studies. Also, the type of fluids consumed and amount of carbohydrates ingested may have influenced fluid retention. Despite individual differences for hydration potential, we calculated that, on average, 15.7% of total fluid intake was lost as urine. A recent randomized trial showed a difference between beverage hydration indexes of commonly used fluids. The article showed that drinks with the highest macronutrient and electrolyte contents—like oral rehydration salts (ORS), orange juice, full fat milk, and skim milk—were the most effective in maintaining fluid balance at rest (27). It is uncertain if the results of this method also apply to fluid retention potential during exercise. Given that most of the runners already consumed highly concentrated gels and bars, and three of them consumed chocolate milk during the race, urine excretion in the current study still equalled 30% of the exercise-induced dehydration. Limiting urine excretion by better fluid retention could therefore potentially help these athletes to maintain their total level of dehydration within a 2–3% range instead of the current 3.6%. Probably as a result of logistic challenges or impossibilities, most research groups reported only acute changes in body weight during exercise to estimate sweat rates and perturbations in hydration status without correcting for urine losses (20). This study adds to our knowledge that neglecting correcting for urine losses will overestimate sweat rate modestly (~5–10%).

Carbohydrate intake is associated with greater endurance performance and capacity when compared with water alone (28). Additionally, higher carbohydrate intakes are associated with better performance. Therefore, ultra-endurance athletes are advised to consume up to 60–90 g of carbohydrates per hour (29, 30). Carbohydrate intake of 90 g/h or higher was reported earlier in individuals (17, 19), but in general, athletes consume lower amounts, which still allows them to finish the race (10–13, 16, 17). During the present study, two of the runners met the recommendation of 60 g/h (intakes of 62.6 and 62.5 g/h). The other three runners reported intakes between 27.1 and 43.7 g/h. Stellingwerff (19) estimated that an ~60 kg world-class elite runner was oxidizing ~3,250 kcal over a 100 km race; his estimation was based on glucose and glycogen representing ~59% of the total metabolized energy (19). They also estimated that glycogen reserves would represent a maximal energy value of ~2,000 kcal for a person of this weight. In the current study, we did not assess pre-race glycogen reserves or the pre-race diet, but only two out of five runners reported to adjust carbohydrate intake in the days before the race. Habitual carbohydrate intake in this type of runners is not necessarily high (17). Although speculative, we hypothesize that the glycogen storage potential should at least be equal in the current group of runners. Extrapolating the estimations of Stellingwerff (19) to the current race situations over 120 km, we speculate that runners should have an exogenous carbohydrate need of at least 375 g (~30–38 g/h), which 4 out of 5 runners exceeded. The only runner observed with a lower carbohydrate intake was actually focusing on running the race with as few carbohydrates as possible with the idea of optimizing fat oxidation as much as possible. Although this person during the first 60 km consumed a very low amount of carbohydrates, he substantially increased his carbohydrate intake in the second part of the race. In the end, the runner consumed a total of 327 g of carbohydrates, which was lower than the estimated need during this type of race. However, it must be taken into account that the glycogen reserves might not be optimized before the race. Overall, runners did not meet the high 90 g/h recommendation. Most of them reported a constant hourly intake, and acknowledging that this type of exercise might actually have a lower carbohydrate need than frequently suggested by Stellingwerff (19), the consumed amount of carbohydrates over the course of the race was probably sufficient.

Several potential factors influenced food and fluid intake. First, runners were accompanied by a cyclist that could hand out all preferred products and drinks at the runner's request. This may not mimic typical food and fluid availability during other races, however, it provided an unique opportunity to get insight into food and fluid intake during an ultra-endurance event without being limited by the amount of aid stations. Secondly, it was relatively cold on the day of the race. We do not believe that the temperature influenced their original fluid intake strategy, as consumption was still within the normal range of previous reports (10–13, 16, 17). Third, the environmental conditions impacted the timing of food intake and fluid excretion because runners entered the four beach sections without their cyclists. In all cases, runners were provided with one or more products before they entered the beach (total beach sections covered 24.5 km). Runners covered these sections within 40–60 min. This time without the cyclist therefore limited their regular feeding opportunity, as runners on average consumed food and/or fluid every 20 min. Finally, food and fluid intake might be affected by perceived GI complaints, as suggested by the literature (24). Fewer complaints were reported during the race, which suggests that there is no specific indication for a relation between running, food and fluid intake and GI complaints. We were able to compare the reported GI complaints during the race and the self-reported GI complaints afterwards using a questionnaire. The incidence and intensity of scored complains differed between both methods, in which the GI complaints during the race are perceived as less severe compared to the GI complaints reported after the race. Further research is necessary to investigate the relevance of this difference in reported GI complaints.

The strength of this study is the use of a continuous observational method to describe timing of carbohydrate and fluid intake during an ultra-endurance event. However, some limitations should be taken into account. First, one runner had a fecal stop and a second runner urinated one time on the beach. In both cases, we made a small correction to our calculation. Normally, gastrointestinal tract losses are small (~100–200 mL/day) (20). In the case of the missing urine sample, we included the average urine excretion of this individual's other samples in the calculation. Second, we had a high drop-out rate (44%), which may have affected our overall results. A previous study, with a drop out race of 63%, described no difference between carbohydrate intake between finishers and non-finishers in a 161 km foot race (12).

In conclusion, runners were able to maintain a relatively constant hourly timing of food and fluid intake during the competition. The total level of dehydration exceeded the current recommendation (<2% dehydration over the course of the race). Products optimizing fluid retention in combination with a slightly higher absolute fluid intake should be considered for future races. Although none of the runners met the literature's suggested high carbohydrate recommendation of 90 g/h, these runners potentially have a lower carbohydrate need, but at least 2 out of 5 runners should consider raising their carbohydrate intake above 30 g/h during future competitions.

## Author contributions

The study was designed by FW, DH, JV, NvdB, and KL. Data were collected by FW, DH, JV, NvdB, KL, and CB. The interpretation of data and preparation of the manuscript were done by FW, DH, and CB. All authors approved the final manuscript.

### Conflict of interest statement

The authors declare that the research was conducted in the absence of any commercial or financial relationships that could be construed as a potential conflict of interest.
